# Pangenomic characterization and grain-development expression analysis of the OVATE gene family in sorghum

**DOI:** 10.3389/fpls.2026.1926574

**Published:** 2026-09-17

**Authors:** Yukun Gao, Puyuan Yang, Yongliang Han, Weiping Yang, Xiaodong Xiang, Jinhua Chang

**Affiliations:** 1 College of Agronomy, Hebei Agricultural University, Baoding, China; 2 North China Key Laboratory for Crop Germplasm Resources of Education Ministry, Hebei Agricultural University, Baoding, China; 3 Handan Academy of Agricultural Sciences, Handan, China; 4 Key Laboratory of Biodiversity Conservation and Sustainable Utilization in Mongolian Plateau for College and University of Inner Mongolia Autonomous Region, College of Life Science and Technology, Inner Mongolia Normal University, Hohhot, China

**Keywords:** *Sorghum bicolor*, OFP gene family, pan-genome analysis, grain development, transcriptional regulation, hormone signaling

## Abstract

The OVATE family proteins (OFPs) are plant-specific regulators that modulate organ morphogenesis and grain development. To elucidate their roles in sorghum (*Sorghum bicolor* L.) grain development, we performed a pan-genome-informed comparative analysis of the OFP family using 17 published sorghum genomes and identified 498 *SbOFP* genes. These 498 genes were classified into 35 *SbOFP* orthologous gene families, which were grouped into 5 Core, 9 Softcore, and 21 Shell families based on presence/absence variation (PAV). Collinearity and *Ka/Ks* analyses showed that 2 Softcore (OFP7, OFP13) and 3 Shell (OFP25, OFP26, OFP30) members exhibited elevated pairwise Ka/Ks ratios in subsets of inter-accession comparisons, whereas the majority of *SbOFPs* evolved under purifying selection during sorghum evolution. Core OFP genes were consistently retained in all 17 accessions (at least one copy per genome) and exhibited higher sequence stability than Shell members. *SbOFP* genes were predominantly expressed during seed and inflorescence development. In addition, *SbOFP* promoters contain abundant hormone-responsive and grain development-related cis-acting elements. Exogenous hormone treatment further showed that SbOFP genes exhibited expression changes in response to ABA, BR, MeJA, and IAA, and that the response patterns were broadly consistent with the presence of corresponding hormone-responsive cis-elements in their promoters. Grain transcriptome analysis identified *SbOFP14* and *SbOFP15* as dominantly expressed members throughout grain development. Weighted gene co-expression network analysis revealed hub *SbOFP* genes co-expressed with NAC, MYB, MADS, and bHLH transcription factors, among which *SbOFP32* and *SbOFP13* showed the highest connectivity. These transcription factors are candidate partners whose expression covaries with that of SbOFP hub genes during grain development. These findings provide the theoretical basis for further elucidating the molecular mechanism of *SbOFP* genes in sorghum grain development and hormone signal regulation, and lay the foundation for genetic improvement of sorghum grain traits.

## 1 Introduction

OVATE Family Proteins (OFPs) are a class of plant-specific transcriptional regulators characterized by a conserved OVATE domain of approximately 70 amino acids at their C-terminus (Snouffer et al., 2020; Wang et al., 2016). Through near-isogenic lines (NILs) in tomato, the key gene controlling fruit shape was mapped and identified as OVATE for the first time. It was first demonstrated that a point mutation in this gene introducing a premature stop codon, changes fruit shape from round to pear-shaped (Liu et al., 2002). Genome-wide identification of OFP genes has been conducted in several plant species, including *Arabidopsis thaliana*, *Oryza sativa*, and *Glycine max*, revealing that OFP family members lack classical DNA-binding domains and cannot directly bind to the promoter regions of target genes (Wang et al., 2011; Ahmad et al., 2023; Zhan et al., 2025). Consequently, these proteins exert their transcriptional repressor functions indirectly by forming regulatory complexes with DNA-binding transcription factors, including TALE (Three Amino acid Loop Extension) homeodomain proteins such as KNOX (KNOTTED-like homeobox) and BELL proteins, as well as Aux/IAA (Auxin/Indole-3-Acetic Acid) proteins (Liu et al., 2014; Wang et al., 2007). Through these protein-protein interactions, OFPs function as versatile transcriptional repressors to orchestrate diverse biological processes, ranging from cell elongation and secondary cell wall formation to ovule development and fruit morphogenesis (Snouffer et al., 2020). Additionally, OFPs mediate plant responses to hormonal signals and environmental stresses, and have emerged as critical genetic factors regulating yield-related traits such as grain size, shape, and weight in crops (Snouffer et al., 2020).

In *Arabidopsis*, 18 *AtOFP* members have been characterized with roles in cell elongation, secondary cell wall formation, ovule development, and brassinosteroid signal transduction (Wang et al., 2011). AtOFP1 localizes to the nucleus and acts as an active transcriptional repressor, negatively regulating cell elongation by suppressing the expression of *AtGA20ox1*, a key enzyme in gibberellin biosynthesis (Wang et al., 2007). And, OFP1/OFP4 interacts with TALE proteins, where TALE proteins provide DNA-binding specificity and OFPs provide repression activity (Li et al., 2011; Liu and Douglas, 2015). OFP proteins can also interact with various transcription factors such as TCP, KNOX, BELL, and ethylene response factors (EIN3/EIL family), forming a complex regulatory network that coordinates organ morphogenesis and hormone signal transduction (Liu et al., 2015; Hackbusch et al., 2005; Snouffer et al., 2020). The role of this gene family in fruit or grain development has been gradually elucidated (Ahmad et al., 2023; Zhan et al., 2025). In rice, 31 *OsOFP* genes have been identified, with individual members regulating distinct agronomic traits (Ahmad et al., 2023). OsOFP2 alters hormonal homeostasis and vasculature development, and its overexpression represses gibberellin biosynthesis, resulting in reduced plant height and altered grain development (Schmitz et al., 2015). OsOFP6 confers resistance to drought and cold stresses by modulating oxidative stress responses and water metabolism (Ma et al., 2017). In addition, OsOFP1 and OsOFP3 participate in brassinosteroid-mediated regulation of plant architecture and grain morphology (Xiao et al., 2017, Xiao et al., 2020). Additionally, OFP family members in *Brassica napus* and *Glycine max* also exhibit extensive functional diversity (Zhan et al., 2025; Liu et al., 2022).

Sorghum (*Sorghum bicolor* L.) ranks as the fifth most important cereal crop worldwide, with an annual production of approximately 60 million tons, providing staple food for over 300 million people (Srivastava et al., 2025). Beyond its role in food security, sorghum holds high economic value in animal feed, brewing industries, and bioenergy production (McCormick et al., 2018). The crop exhibits exceptional drought tolerance, salinity resistance, and high water use efficiency, making it a critical strategic crop for mitigating global climate change and water scarcity (Wang et al., 2026). With a relatively compact genome size of approximately 700–800 Mb and its status as a diploid C_4_ plant belonging to the Panicoideae subfamily, sorghum has emerged as an ideal model organism for functional genomics research among cereal crops (Morris et al., 2026). The availability of over 45,000 accessions in global germplasm collections further enhances its value for genetic and genomic studies. With the advance of sorghum pan genome project, comparative genomic research based on multiple strains (such as BTx623, RTx430, Rio, and a variety of local varieties and wild relatives) has been carried out in depth (Paterson et al., 2009; Deschamps et al., 2018; Cooper et al., 2019; Tao et al., 2021). The pan genome can fully reveal the presence absence variation (PAV) of genes, which greatly expands our understanding of genetic diversity and functional differentiation of gene families. Existing studies have largely relied on single or limited genomic resources, failing to fully uncover the genetic diversity of OFP genes in sorghum germplasm. Systematic studies of the OFP gene family remain limited, particularly regarding their functional mechanisms in hormone signaling and grain development.

As a significant crop supporting food security and economic systems, sorghum requires optimized yield. Grain development is a critical determinant of both yield potential and grain quality, with hormonal regulation serving as a central hub coordinating these processes. The 17 sorghum accessions used in this study represent diverse genetic resources, including the reference genome line BTx623, sweet sorghum Rio, Chinese elite varieties (Ji2731and Ji2055), restorer line (RTx430), ICRISAT germplasm collections (IS series), landraces with margaritiferum characteristics (PI series), wild relatives (*S. propinquum*, S369-1), and geographically isolated ecotypes. This panel spans major sorghum races and captures the domestication gradient from wild progenitors to improved cultivars (Tao et al., 2021; Wei et al., 2024). A previous study identified 26 OFP genes in the BTx623 genome (An et al., 2024). Here, we extended the analysis to the pan-genome level across 17 sorghum accessions, and integrated grain-development transcriptome and exogenous hormone treatment data to further investigate the roles of SbOFPs in grain development.

In this study, we conducted a pan-genome-informed comprehensive analysis of the sorghum OFP gene family across these 17 accessions. We investigated their phylogenetic relationships, gene structures, conserved motifs, physicochemical properties of proteins, and chromosomal locations. Cis-acting elements in promoter regions were analyzed to clarify hormone-responsive and functional elements. Gene expression characteristics under hormone treatments were examined using Reverse Transcription Quantitative PCR (RT-qPCR). We performed collinearity analysis and calculated *Ka/Ks* ratios across 17 accessions to assess selective pressures and evolutionary features. Furthermore, gene expression data for OFP genes were extracted from multiple tissues and grain developmental stages. The weighted gene co-expression network was constructed to identify the functions of OFP genes in grain development and their associated regulatory genes. These results establish a systematic characteristics of the sorghum OFP family, providing candidate gene resources inventory for future functional studies and molecular breeding. This study advances our understanding of the functional and evolutionary dynamics of OFP transcriptional regulators in grain crops.

## 2 Materials and methods

### 2.1 Data source, sequence retrieval, physicochemical properties analysis

The genome assemblies, genome sequences, protein sequences, and corresponding annotation files for 17 sorghum accessions were obtained from the Phytozome, Ensembl Plants, and the China National GeneBank (CNGB) (**Supplementary Table 1**). Detailed download information for the accessions used in this study is provided in **Supplementary Table 1**. These accessions represent diverse genetic backgrounds, encompassing 1 wild relative species (*Sorghum propinquum*, S369-1), 1 wild subspecies (*Sorghum bicolor* ssp. *verticilliflorum*, AusTRCF), 2 accessions of *S. bicolor* ssp. *bicolor* var. *margaritiferum* (PI525695 and IS19953), 3 weedy accessions of *S. bicolor* ssp. *drummondii* (353, PI536008, and PI532566), and 10 cultivated sorghum accessions (*S. bicolor* ssp. *bicolor*) (Ji2731, IS929, IS3614-3, IS8525, Rio, IS12661, R931945-2-2, RTx430, Ji2055, and BTx623) (Tao et al., 2021; Wei et al., 2024). The genome sequence and annotation of Arabidopsis thaliana were retrieved from the TAIR database (https://www.arabidopsis.org/).

A phylogenetic tree was constructed based on the pan-genome data of 17 sorghum accessions. Single-copy orthologous genes were identified using OrthoFinder (v2.3.1) with default parameters (Emms and Kelly, 2019). The aligned protein sequences were then converted to codon-based nucleotide alignments using PAL2NAL (Suyama et al., 2006). A maximum likelihood phylogenetic tree was reconstructed using IQ-TREE (v2.2.0) with 1,000 ultrafast bootstrap replicates and the best-fit substitution model determined by ModelFinder (Minh et al., 2020). The resulting tree was visualized and annotated using FigTree (v1.4.4).

To identify OFP-encoding genes, we employed a dual-strategy approach combining HMM profile search and transcription factor annotation. The hidden Markov model (HMM) profile of the OVATE domain (PF07107) was downloaded from the Pfam database (http://pfam.xfam.org/) (Mistry et al., 2021). All predicted protein sequences were subjected to domain searches using the hmmsearch program from the HMMER suite (v3.0) with default parameters. Simultaneously, all protein sequences were analyzed using iTAK software (v2.0.1) for the classification of transcription factors and transcriptional regulators, the latter including the OFP family (Zheng et al., 2016). Candidate genes identified by either approach were merged, and we further validated putative OFP-encoding genes using the NCBI Batch CD-Search tool (https://www.ncbi.nlm.nih.gov/cdd/) to confirm the presence of the OVATE domain (Marchler-Bauer et al., 2017), with manual inspection of alignment coverage (>70%) and identity (>80%) for each hit.

Only genes containing the complete OVATE domain were retained as authentic OFP family members. We analyzed the physicochemical properties of each OFP protein, including amino acid length, molecular weight, isoelectric point (pI), instability index, aliphatic index, and hydropathicity, using the ExPASy ProtParam tool (https://web.expasy.org/protparam/), and conducted subcellular localization predictions using Plant-mPLoc (Chou and Shen, 2010). Differences in physicochemical properties among the Core, Softcore, and Shell categories were assessed using the two-sided Wilcoxon rank-sum test, with Benjamini-Hochberg correction for multiple comparisons. To account for non-independence among alleles of the same orthogroup, a sensitivity analysis was performed using one representative (BTx623 allele) per orthogroup (n = 35).

### 2.2 Pan-genome-based classification and tissue expression of OFP gene families

We performed gene family clustering across the 17 sorghum accessions to identify Core, Softcore, Shell, and Private OFP gene families. Homology searches of all protein sequences were conducted using BLASTP with an E-value threshold of 1×10^-5^. We applied OrthoFinder (v2.3.1) with an inflation parameter of 1.5 to group protein sequences into homologous and orthologous gene families (Emms and Kelly, 2019). We defined gene families present in all 17 accessions (17/17) as Core gene families, those shared by 16 accessions (16/17) as Softcore gene families, those shared by 2–15 accessions (2-15/17) as Shell gene families, and those unique to a single accession (1/17) as Private gene families. We constructed the presence/absence variation (PAV) matrix of OFP genes across the 17 accessions based on OrthoFinder clustering results and manual curation of BLAST hits. We determined copy number variation (CNV) by counting gene copy numbers (0, 1, 2, or 3) in each accession, with genes showing copy number ≥2 in at least one accession classified as CNV genes.

To investigate tissue-specific expression patterns of *SbOFP* genes, we retrieved RNA sequencing data for 13 tissues from the Sorghum Genome Multi-omics Database (SGMD, https://sorghum.genetics.ac.cn/SGMD/Expression.html) (Chen et al., 2025). The dataset encompasses roots, seedlings, leaves (four developmental stages: Leaf 1-4), stems (two developmental stages: Stem 1-2), inflorescences (two developmental stages: Inflorescence 1-2), and seeds (three developmental stages: Seed 1-3). Expression levels were quantified as transcripts per million (TPM) values. We analyzed tissue-specific expression patterns of *SbOFP* genes by extracting TPM values for all OFP family members across the 13 tissues and visualized the results using heatmaps generated in R (v4.0) with the pheatmap package (Kolde, 2025). For orthogroups containing multiple BTx623 copies, the first copy in chromosomal order was selected as the representative, and its expression value was used in family-level analyses; the locus-to-orthogroup correspondence for all 498 genes is provided in **Supplementary Table 3**.

### 2.3 Phylogenetic analysis, gene structure, and motif identification of the OFP gene family

Eighteen *AtOFP* genes from *Arabidopsis thaliana* were retrieved from the TAIR database (https://www.arabidopsis.org/), identified based on the results reported by Wang et al. (2011). To construct a representative phylogenetic tree, one SbOFP protein was selected from each of the 35 orthologous families, with the BTx623 allele preferentially selected; when a subfamily contained more than one BTx623 member, the longest protein encoded by the primary transcript was used. A total of 53 protein sequences (35 sorghum and 18 *Arabidopsis*) were aligned using default parameters in MEGA (v12) (Kumar et al., 2024), and a maximum likelihood phylogenetic tree was constructed with 1,000 bootstrap replicates. The resulting tree was visualized using FigTree (v1.4.4, http://tree.bio.ed.ac.uk/software/figtree/).

Conserved motifs in sorghum OFP proteins were identified using the MEME suite (http://meme-suite.org/tools/meme) (Bailey et al., 2015). To assess protein homology between sorghum and Arabidopsis OFP proteins, DIAMOND v0.8.25 was used with parameters --max-target-seqs 5 and --evalue 0.00001 (Buchfink et al., 2015). Based on the genome annotation (GFF) files, the exon–intron structures of the OFP genes were extracted and visualized using TBtools (v2.136) (Chen et al., 2020).

### 2.4 *Ka/Ks* calculation and collinearity analysis

OFP protein and coding sequences from the 17 sorghum genomes were obtained from the sorghum pan-genome dataset. Bulk sequence alignment was performed using ParaAT v2.0 (Zhang et al., 2012), and *Ka/Ks* ratios were calculated with the KaKs Calculator (Zhang et al., 2006). The distribution of *Ka/Ks* values was visualized as a ridge plot using the R packages ggridges and ggplot2 in R (v4.0.3). Chromosomal locations of *SbOFP* genes across the 17 Sorghum genomes were extracted from the corresponding GFF files.

All protein-coding genes from the 17 sorghum accessions were subjected to pairwise BLAST analysis with an E-value cutoff of 1×10^-5^. The BLAST results were processed using MCScanX (v1.1) to identify collinear chromosomal regions both within individual accessions and across different accessions (Wang et al., 2012). For OFP gene family-specific collinearity analysis, BLAST hits in m8 format and General Feature Format (GFF) files for each genome were used as input for the collinearity detection toolkit based on the adjusted MCScan algorithm. The BTx623 genome served as the reference for cross-accession comparisons. Deplicated gene pairs and homologous genome fragments were visualized using TBtools (v2.136) (Chen et al., 2020). This analysis identified segmental duplications, tandem duplications, and syntenic relationships of OFP genes across the 17 sorghum genomes.

### 2.5 Cis-acting element analysis of SbOFP genes

Upstream 2-kb sequences of all 498 SbOFP genes from the 17 accessions were extracted using TBtools (v2.136) (Chen et al., 2020). Cis-acting elements in promoters were predicted using the PlantCARE database (http://bioinformatics.psb.ugent.be/webtools/plantcare/html/) (Lescot et al., 2002). The reported element counts represent values aggregated across the promoters of all 498 SbOFP genes. Classified elements were visualized using the pheatmap v1.0.12 package.

### 2.6 Plant materials and hormone treatments

The reference genome accession BTx623 was used as the experimental material for cultivation and hormone treatments. Seeds were surface-sterilized, sown in nutrient soil, and cultivated in an artificial climate chamber (16 h light/8 h dark, 28 °C). At the three-leaf stage, seedlings with consistent growth were selected for exogenous hormone treatments. Leaves were sprayed with 100 μmol/L 24-epibrassinolide (BR), methyl jasmonate (MeJA), abscisic acid (ABA), or indole-3-acetic acid (IAA) solutions (all from Sigma-Aldrich, St. Louis, MO, USA) (Dugas et al., 2011). Plants sprayed with an equal volume of distilled water served as the blank control. Functional leaves were collected at 0, 3, 6, 12, and 24 h after treatment, with three biological replicates per time point. All samples were immediately frozen in liquid nitrogen and stored at -80 °C.

### 2.7 Total RNA extraction and reverse transcription quantitative PCR analysis

Total RNA was extracted from samples using the RNAprep Pure Plant Kit (Tiangen Biotech, Beijing, China) according to the manufacturer’s instructions. First-strand cDNA was synthesized from 1 μg of total RNA using the PrimeScript™ RT Reagent Kit with gDNA Eraser (TaKaRa, Dalian, China) according to the manufacturer’s protocols. Six *SbOFP* genes (*SbOFP1*, *SbOFP6*, *SbOFP14*, *SbOFP22*, *SbOFP31*, and *SbOFP33*) were selected for RT-qPCR expression analysis. The sorghum *SbActin1* (*BTx623.01G123700*) gene was used as the internal reference for normalization. The specific primer sequences used for RT-qPCR are listed in **Supplementary Table 2**. RT-qPCR was performed on a BIO-RAD CFX96 Touch Real-Time PCR System (Bio-Rad Laboratories, Hercules, CA, USA) using TB Green^®^ Premix Ex Taq™ II (TaKaRa, Dalian, China). Three technical replicates were included for each biological replicate. The relative expression levels of target genes were calculated using the 2^^-^ΔΔCT^ method, with 0 h treatment samples as the control (Schmittgen and Livak, 2008). Statistical analysis was performed using SPSS 26.0 with two-way ANOVA, followed by Tukey’s HSD test for multiple comparisons (*p* < 0.05).

### 2.8 Field experiment and transcriptome analysis

To investigate the expression patterns of *SbOFP* genes during grain development, two sorghum varieties, HN16 and QS, were planted at the experimental farm of Hebei Agricultural University, Baoding, China, in the spring of 2025. A randomized plot design was used, with three replicate plots per variety (30 m² per plot; 30×50 cm spacing). Plants were grown under standard field management practices with regular irrigation, fertilization, and pest control. Grain length and grain width of the two varieties were measured at maturity (**Supplementary Table 6**). At anthesis, panicles were tagged to ensure synchronized sampling, and developing grains from the middle third of the panicle were collected at 8, 14, and 28 days after anthesis (DAP) to minimize positional effects within the inflorescence. Grain length (GL) and grain width (GW) were measured using a digital caliper (0.01 mm precision) at each sampling stage. Ten representative grains per replicate were measured, and mean values were calculated.

For each variety and developmental stage, three independent biological replicates were harvested, with each replicate consisting of pooled grains from five randomly selected plants grown under identical field conditions. Total RNA was extracted from developing seeds using TRIzol reagent (Invitrogen). After quality assessment with NanoDrop and Agilent 2100 Bioanalyzer, strand-specific RNA-seq libraries were constructed using the NEBNext Ultra RNA Library Prep Kit (NEB, USA) and sequenced on the Illumina NovaSeq 6000 platform (150 bp paired-end). Sequencing yielded an average of 42.5 million clean reads per sample (Q30 > 97.6%), with an average of 85.5% of clean reads uniquely mapped (**Supplementary Table 7**).

Raw reads were processed with Trimmomatic (v0.39) (Bolger et al., 2014), mapped to the BTx623 reference genome using HISAT2 (v2.2.1) (Kim et al., 2019), and gene counts were quantified using StringTie (v2.1.4), from which TPM values were derived for expression visualization. Differential expression analysis was performed with DESeq2 using the raw count data (|log_2_FC| ≥ 1, adjusted *p* < 0.05) (Love et al., 2014). The identification of core and variable OFP expression across grain developmental stages was based on integrated transcriptome data from both varieties. Gene Ontology (GO) enrichment analysis was performed using the GOseq R package, which accounts for gene length bias in RNA-seq data, with *q-value* ≤ 0.05 as the significance threshold (Young et al., 2010). Gene-specific primers for differentially expressed genes (DEGs) were designed using Primer Premier 5.0 (**Supplementary Table 2**). To validate the expression levels of 6 differentially expressed genes (DEGs), RT-qPCR was performed following the protocol described in 2.7.

### 2.9 Prediction and analysis of potential target genes and protein interactions of OFPs

Weighted Gene Co-expression Network Analysis (WGCNA) was performed using the WGCNA R package (v1.72) based on grain developmental transcriptome data (Langfelder and Horvath, 2008). The expression matrix was preprocessed by filtering genes with low variation (median absolute deviation [MAD] < 0.05) and excessive missing values (>20% of samples), and 20,120 genes were retained for network construction. The soft-thresholding power (β) was determined using the pickSoftThreshold function with R² ≥ 0.9 as the scale-free topology criterion (Langfelder and Horvath, 2008). Co-expression modules were identified using the blockwiseModules function with a signed network (soft-thresholding power β = 8, selected with R² ≥ 0.9; minModuleSize = 30; deepSplit = 2; mergeCutHeight = 0.3). Module eigengenes were correlated with grain phenotypic traits (grain length and grain width), and modules significantly associated with grain traits (|Pearson r| > 0.7, *p* < 0.01) were identified as candidate modules. Hub genes within trait-related modules were identified based on intramodular connectivity and gene significance.

To construct a multilayered hierarchical gene regulatory network (ML-hGRN) for OFP-mediated grain development, the bottom-up graphic Gaussian model (GGM) algorithm was employed (Kumari et al., 2016). This method places target genes at the bottom layer and evaluates connections between each target gene and candidate regulatory genes. The algorithm retains all connections in which a regulatory gene significantly affects a target gene based on conditional independence testing. The regulatory genes with the highest interaction frequency are retained as the second layer, and the number of genes retained is based on an optimization function. In this study, the inputs for the bottom-up GGM algorithm included *SbOFP* hub genes extracted from the blue module as target genes and differentially expressed transcription factors (TFs) from the grain developmental transcriptome data as candidate regulatory genes. A gene regulatory network was constructed with these *SbOFP* genes as the bottom layer and the associated TFs as the upper layer, and the networks were visualized using Cytoscape (v3.9.0) (Shannon et al., 2003).

## 3 Results

### 3.1 Identification of the OFPs in sorghum and features of Core, Softcore and Shell OFPs

A total of 498 OFP genes were identified across 17 sorghum accessions using iTAK and HMMER software. The number of OFP genes per accession ranged from 19 (AusTRCF) to 36 (BTx623), with each accession containing a mean of 29 OFP-encoding genes (**Figure 1**; **Table 1**; **Supplementary Table 3**). Based on the presence/absence variation (PAV) patterns across the 17 accessions, the 35 OFP gene families were classified into three categories: Core (present in all 17 accessions), Softcore (present in 16 accessions), and Shell (present in 2–15 accessions) (**Figure 1A**). Five (OFP2, OFP3, OFP4, OFP5, and OFP6) were classified as Core gene families, comprising 85 genes (17.1%). And, OFP4, OFP5, and OFP6 were strictly single-copy across all accessions, whereas OFP2 and OFP3 showed accession-specific duplications. The Core OFPs showed high sequence conservation with average amino acid identity of 96.5% across accessions. Nine families were classified as Softcore gene families, comprising 144 genes (28.9%) (**Figure 1B**). The remaining 21 families were classified as Shell gene families, comprising 269 genes (54.0%). Among these, OFP1, OFP9, and OFP31 exhibited copy number variation (CNV), with 2–3 paralogous copies detected in certain accessions (**Figure 1A**), indicating duplication and expansion events. No gene families were found to be present in only a single accession.

**Figure 1:**
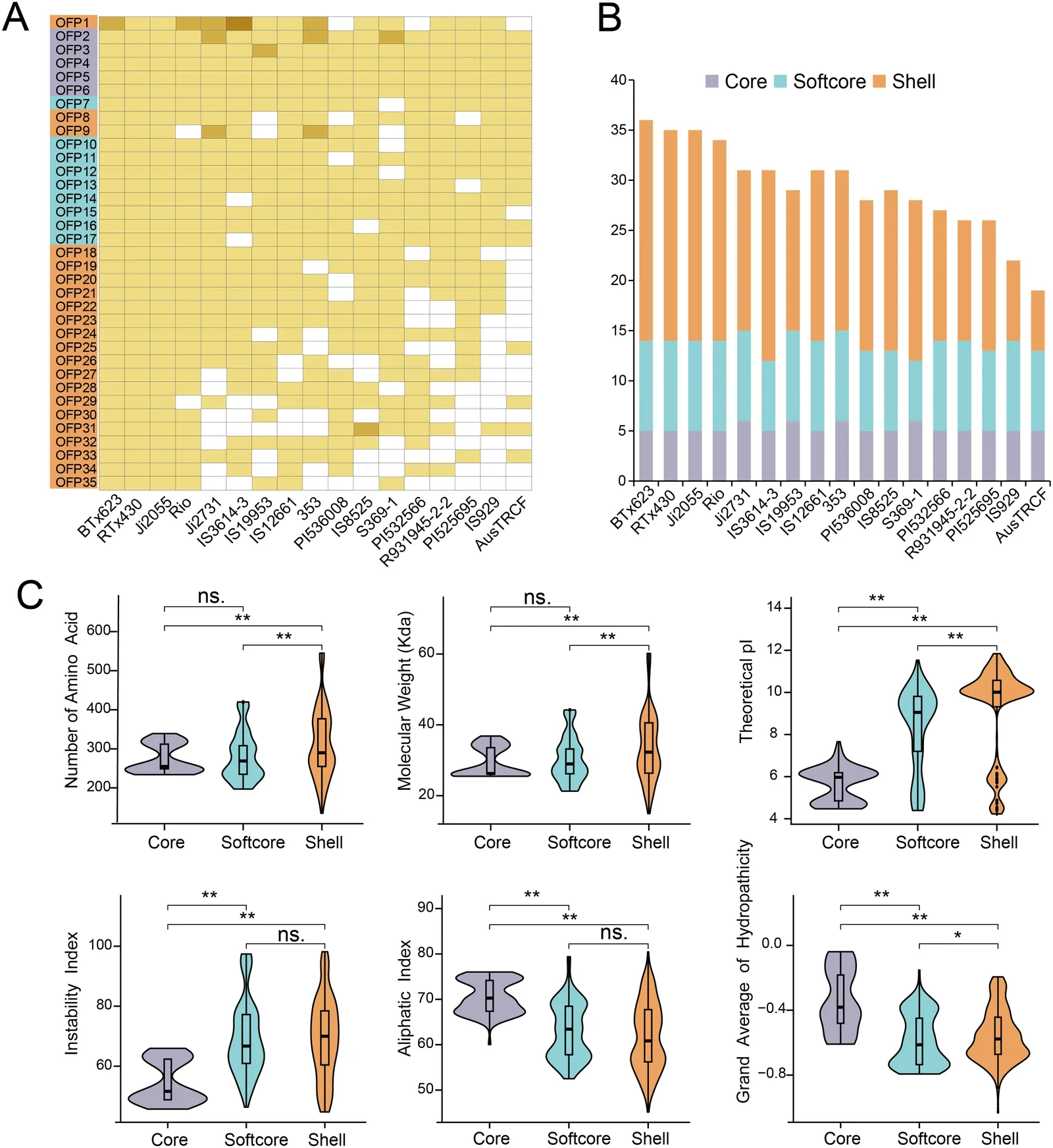
Pan-genomic classification and physicochemical properties of the OFP gene family. **(A)** Presence/absence variation (PAV) heatmap of 35 OFP gene families across 17 sorghum accessions. Light yellow indicates one copy, brown indicates two copies, dark brown indicates three copies, and white indicates absence. **(B)** Number of Core, Softcore, and Shell OFP genes in each accession. **(C)** Comparing physicochemical properties among Core, Softcore, and Shell proteins, including protein length (aa), molecular weight (kDa), theoretical isoelectric point (pI), instability index, aliphatic index, and grand average of hydropathicity. Significance was determined by two-sided Wilcoxon rank-sum test with Benjamini-Hochberg correction: **adjusted *p* < 0.01; *adjusted *p* < 0.05.

**Table 1:** Physicochemical characteristics of OFP proteins in 17 Sorghum accessions.

Accession	Gene number	Protein length (aa)			Molecular Weight (kDa)			Theoretical pI			Instability Index			Aliphatic Index			Grand average of hydropathicity		
		Min	Max	Mean	Min	Max	Mean	Min	Max	Mean	Min	Max	Mean	Min	Max	Mean	Min	Max	Mean
BTx623	36	197	543	308.03	21.54	60.06	33.03	4.49	11.62	8.47	47.77	94.36	67.4	49.55	75.1	62.81	-0.78	-0.06	-0.53
353	31	177	420	285.29	19.63	44.22	30.76	4.52	11.14	8.39	47.66	98.15	66.42	52.76	75.1	64.89	-0.77	-0.06	-0.53
AusTRCF	19	197	416	291.32	21.29	44.55	31.48	4.52	10.72	7.96	47.16	93.95	65.34	55.43	75.1	64.98	-0.78	-0.06	-0.55
IS12661	31	200	543	305.77	20.82	60.06	32.89	4.48	11.62	8.48	44.76	95.63	68.32	49.74	75.15	63.08	-0.77	-0.07	-0.54
IS19953	29	170	540	301.93	17.94	60.01	32.35	4.39	11.6	8.29	45.92	98.15	66.8	50.25	75.69	63.71	-0.77	-0.05	-0.52
IS3614-3	31	177	545	285.26	19.63	60.17	30.67	4.46	11.62	8.34	45.95	95.74	67.14	51.19	75.1	63.41	-0.77	-0.07	-0.53
IS8525	29	178	543	306.59	19.73	60.06	33.00	4.44	11.6	8.56	45.02	96.93	69.13	49.55	76.1	63.14	-1.03	-0.06	-0.56
IS929	22	177	428	287.36	19.63	44.18	30.88	4.45	11.05	7.87	46.24	97.39	64.11	52.76	80.46	64.46	-0.78	-0.09	-0.52
Ji2055	35	200	543	311.91	21.39	60.06	33.46	4.49	11.6	8.44	47.77	94.36	67.87	49.55	75.1	63.11	-0.78	-0.06	-0.53
Ji2731	31	177	543	295.61	19.63	60.06	31.85	4.5	11.6	8.35	46.04	95.63	65.9	49.55	75.2	63.28	-0.77	-0.07	-0.55
PI525695	26	136	542	279.88	15.03	59.59	30.13	4.46	11.59	8.22	45.66	94.8	66.47	49.56	75.34	64.05	-0.76	-0.06	-0.52
PI532566	27	156	423	285.48	16.45	44.22	30.60	4.23	11.45	7.86	46.37	95.82	66.84	49.56	76.02	63.81	-0.89	-0.06	-0.51
PI536008	28	207	534	306.93	21.39	58.94	33.01	4.4	11.59	8.06	46.58	98.15	68.84	50.26	79.38	64.38	-0.8	-0.07	-0.52
R931945-2-2	26	177	428	290.04	19.63	44.22	31.12	4.48	10.97	7.93	44.76	95.63	65.42	49.55	75.1	62.83	-0.78	-0.06	-0.52
Rio	34	177	543	297.62	19.63	60.06	31.94	4.47	11.6	8.27	47.77	94.36	67.34	45.41	79.38	63.46	-0.83	-0.06	-0.52
S369-1	28	135	534	303.71	14.95	55.76	32.56	4.52	11.84	8.29	45.71	92.97	64.33	45.16	79.38	64.53	-0.87	-0.04	-0.5
RTx430	35	200	543	308.54	21.66	60.06	33.10	4.43	11.6	8.41	46.16	98.15	67.63	51.19	75.1	63.05	-0.78	-0.06	-0.53

The length of OFP protein sequence ranged from 135 to 545, with an average of 298, with the shortest observed in CCG016153 (S369-1, OFP22) and the longest in CCG012490 (IS3614-3, OFP23) (**Table 1**; **Figure 1A**). The molecular weight (MW) of the proteins ranged from 14.95 kDa to 60.06 kDa, averaging 33.28 kDa, and the predicted pI varied from 4.23 to 11.84, with an average of 8.56 (**Table 1**). Instability index (II) revealed that 462 proteins (92.77%) had an II value greater than 40, suggesting that most OFPs are predicted to be unstable proteins (**Table 1**). Moreover, the grand average of hydropathicity (GRAVY) values for all OFP proteins was below 0, indicating that they are hydrophilic. Subcellular localization predictions from Plant-mPLoc showed that 84.14% (419/498) of OFP proteins were predicted to localize to the nucleus, 8.43% (42/498) in both chloroplast and nucleus, 4.42% (22/498) in both cell membrane and nucleus, 2.81% (14/498) in chloroplast, and 0.20% (1/498) in both mitochondrion and nucleus (**Supplementary Table 4**). 484 OFP proteins (97.2%) were predicted to localize, at least in part, to the nucleus, suggesting that the nucleus is the primary functional compartment (Wang et al., 2007).

Core, Softcore, and Shell OFP families exhibited significantly distinct physicochemical properties. Compared to Core and Softcore, Shell OFP proteins displayed significantly larger protein sizes and higher molecular weights (*p* < 0.01) (**Figure 1C**). The theoretical pI ranged from acidic in Core proteins (median ~6) to basic in Softcore and Shell proteins (median 9-10), with significant differences detected among all three categories (*p* < 0.01). Core OFPs demonstrated significantly lower instability indices than Softcore and Shell proteins (*p* < 0.01), suggesting greater predicted structural stability. The aliphatic index was significantly higher in Core than in Softcore and Shell proteins (BH-adjusted *p* < 0.01), consistent with predicted higher thermostability of Core members. Additionally, Core OFPs exhibited significantly less negative grand average of hydropathicity values compared to Softcore and Shell proteins (*p* < 0.01), reflecting relatively higher hydrophobicity. These distinct physicochemical profiles support the functional stratification of sorghum OFP genes, with Core members maintaining conserved structural features and Softcore/Shell members exhibiting greater sequence and property diversification (**Figure 1C**).

### 3.2 Phylogenetic and tissue expression analysis of sorghum *OFP* genes

To elucidate the phylogenetic relationships of OFPs, 35 representative SbOFP protein sequences (one per orthologous family) and 18 *Arabidopsis* orthologs were used for phylogenetic inference. Based on the phylogenetic tree, the OFP family was classified into four subgroups (**Figure 2A**; **Supplementary Figure 1**). SbOFP genes co-clustered with their *Arabidopsis* orthologs in varying proportions within specific subgroups, suggesting conserved homology among OFP genes in sorghum and *Arabidopsis*. Subgroup I encompasses 7 OFP proteins (OFP3, OFP5, OFP6, OFP7, OFP13, OFP19, OFP30) and 3 evolutionarily conserved AtOFP members. Motif 5 is specifically distributed in Subgroup I. Subgroup II encompasses the 5 OFP proteins and 4 AtOFPs orthologs (**Figure 2A**). Category distribution analysis revealed that Subgroup I comprises Core OFP genes (OFP3, OFP5, OFP6), with Core gene proportion predominating in Subgroup II.

**Figure 2:**
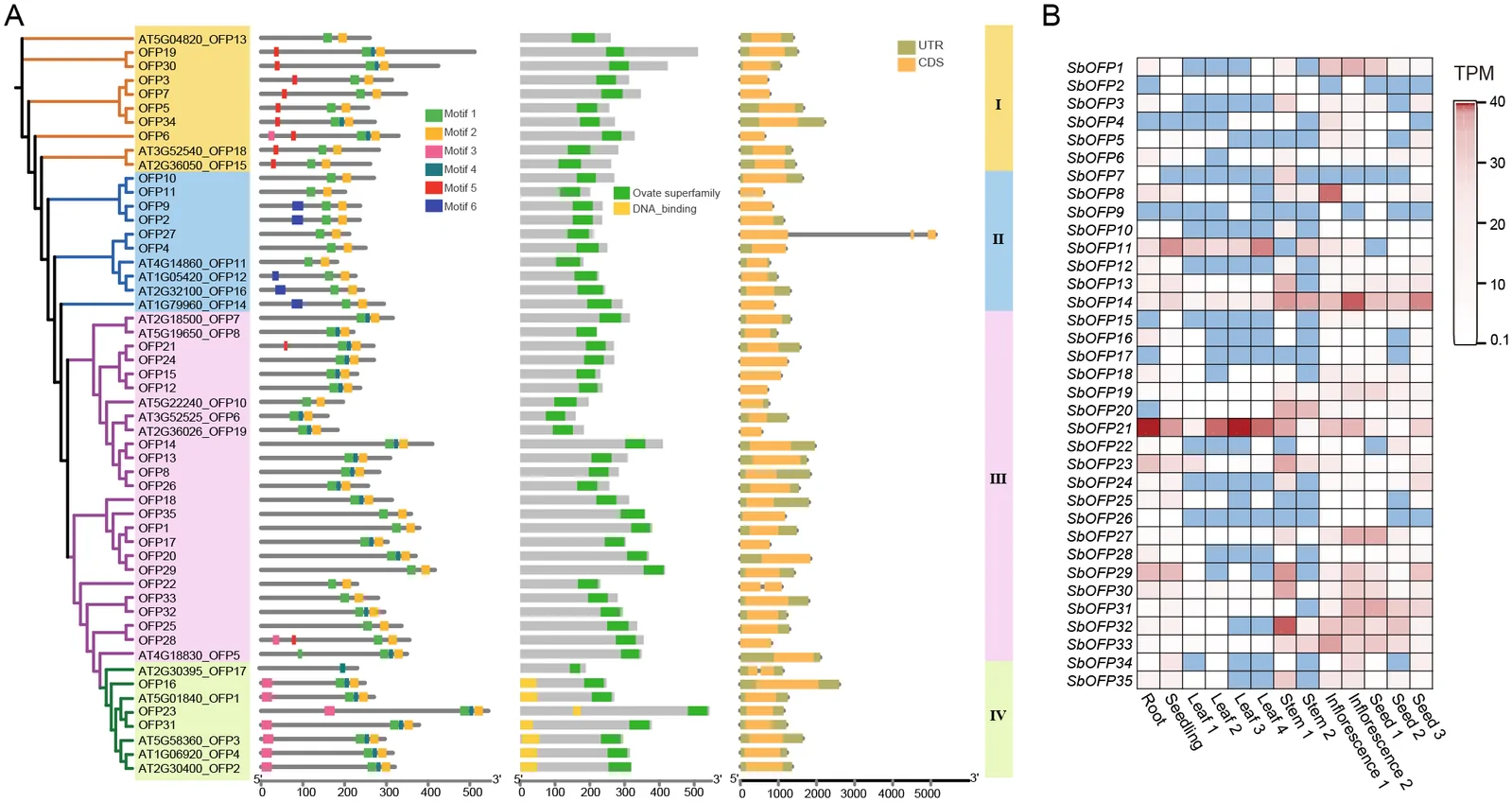
Phylogenetic relationships, conserved motif architecture, and tissue-specific expression patterns of SbOFP genes. **(A)** Phylogenetic tree and conserved motif analysis of OFP family. **(B)** Tissue-specific expression of SbOFP genes in sorghum. Tissues include root, seedling, leaf (1-4), stem (1-2), inflorescence (1-2), and seed (1-3).

Subgroup III contains the largest number of members (19 SbOFPs), constituting an intermediate group with distinctive motif assemblages. Subgroup IV comprises 3 SbOFPs and AtOFP1-AtOFP4 (**Figure 2A**). Motif 3 is abundant in Subgroup IV, indicative of lineage-specific diversification. Both Subgroups III and IV are enriched for Shell OFPs, with Subgroup IV harboring exclusively non-core members (OFP16, OFP23, OFP31). These findings demonstrate that protein-coding sequences are evolutionarily conserved within subgroups, yet exhibit variability in gene structure and motif composition.

To investigate the spatial expression patterns of the *SbOFP* gene family, we examined transcript levels of *SbOFP* representative genes across 13 tissues. The majority of *SbOFP* genes exhibited high expression in reproductive tissues, particularly during seed development and inflorescence formation (**Figure 2B**). *SbOFP19*, *SbOFP27*, *SbOFP31*, *SbOFP17*, *SbOFP14*, *SbOFP1*, *SbOFP8*, *SbOFP4*, and *SbOFP22* all exhibited elevated expression in seed and inflorescence tissues, indicating a role for these SbOFP members in reproductive development. Additionally, *SbOFP35*, *SbOFP32*, *SbOFP30*, *SbOFP10*, *SbOFP7*, *SbOFP13*, *SbOFP3*, and *SbOFP24* exhibited high expression in stem tissues (**Figure 2B**). In contrast, *SbOFP28*, *SbOFP16*, and *SbOFP25* showed relatively higher expression in root and seedling tissues. *SbOFP21* displayed a unique expression pattern distinct from all other family members, with widespread expression across multiple tissues, suggesting that it may participate in diverse regulatory processes. Collectively, sorghum OFP family genes are predominantly associated with reproductive development, particularly seed and inflorescence formation.

### 3.3 OFP gene synteny and selective pressure

Based on phylogenetic analysis of 17 sorghum genomes, the clustering pattern was consistent with the established infraspecific taxonomy of sorghum (Tao et al., 2021). The wild relative *S. propinquum* (S369-1) was positioned as the outgroup. Within *S. bicolor*, two wild subspecies formed branches independent of cultivated accessions, indicating their status as early-diverged lineages. The cultivated subspecies clustered into a single clade, with *S. bicolor* ssp. *bicolor* comprising 10 accessions as the largest group (**Figure 3A**). Through pan-genome synteny analysis of 17 Sorghum genomes, we systematically identified the collinearity and evolutionary relationships among OFP gene family members. Whole-genome synteny revealed that OFP genes exhibited high structural conservation, with the majority residing within highly stable syntenic blocks (**Figure 3B**). Among *S. bicolor* ssp. *bicolor* genomes, strong linear correspondence was observed, with most OFP genes showing one-to-one orthologous distribution, forming a conserved gene set across multiple accessions. In contrast, fewer syntenic connections were detected between *S. bicolor* ssp. v*erticilliflorum* and *S. bicolor* ssp. *bicolor* (*margaritiferum*). These results indicate that duplicated OFP genes were detected less frequently between subspecies than within the same subspecies, reflecting stable gene number and function within subspecies.

**Figure 3:**
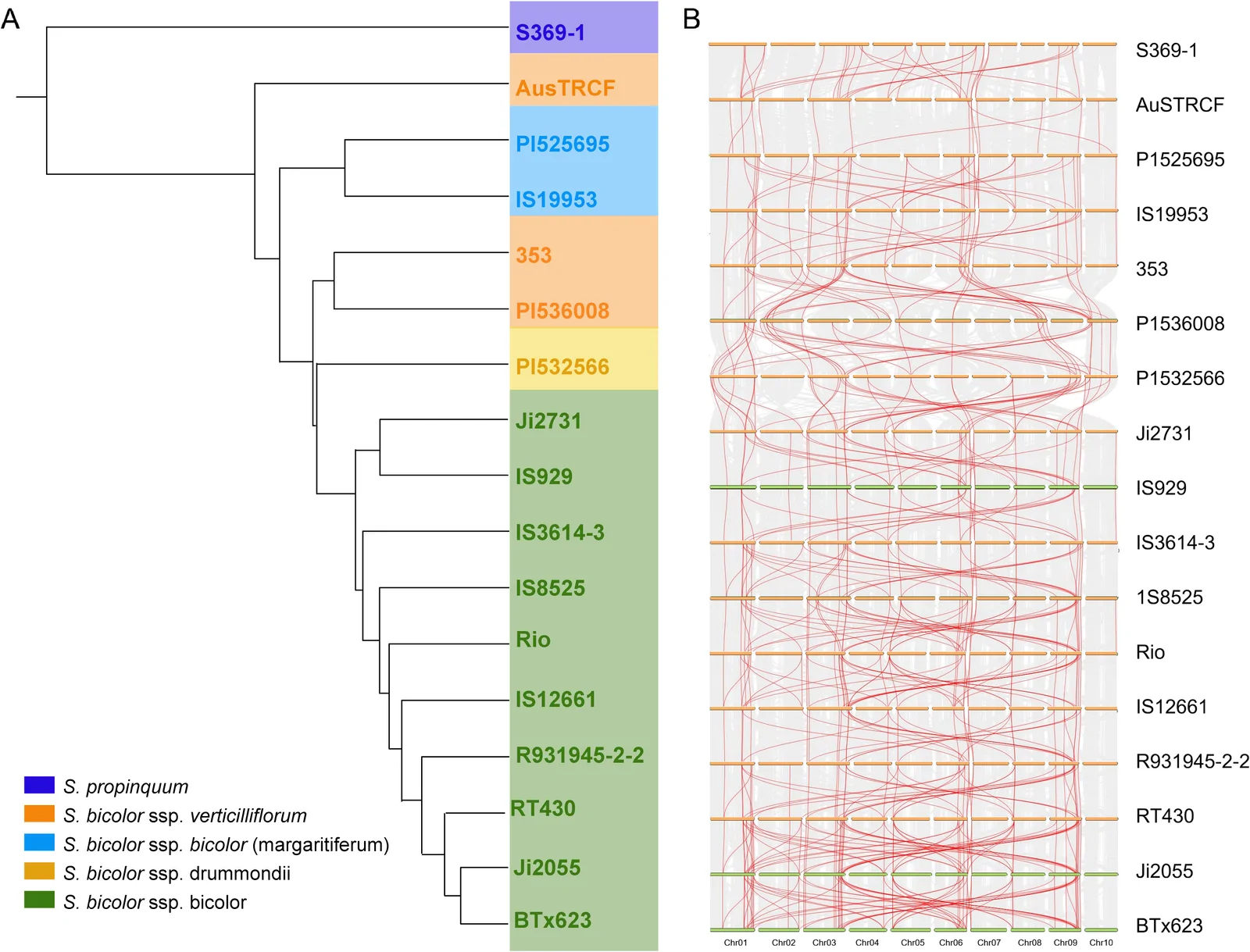
Phylogenetic relationships and genome-wide synteny analysis of 17 sorghum accessions. **(A)** Phylogenetic tree of 17 sorghum accessions based on-genome sequence. **(B)** Genome-wide synteny of 17 accessions. Red lines indicate OFP gene synteny, with chromosomes (Chr01-Chr10) represented as horizontal bars.

The distribution of OFP genes across chromosomes was non-uniform among the 17 sorghum accessions. Chromosomes 1 and 3 harbored a higher number of OFP genes. In BTx623, 36 *SbOFPs* were mapped to ten chromosomes, whereas in AusTRCF, five *SbOFPs* remained unanchored to chromosomes (**Supplementary Figure 2**). To investigate the selective pressures acting on sorghum OFP genes, *Ka/Ks* ratios were calculated for both intra-accession and inter-accession comparisons. *OFP7*, *OFP13*, *OFP25*, *OFP26*, and *OFP30* exhibited multimodal *Ka/Ks* distributions, with 26, 7, 18, 15, and 2 gene pairs, respectively, showing *Ka/Ks* ratios greater than 1, indicating elevated pairwise dN/dS ratios in these pairwise comparisons (**Figure 4A**; **Supplementary Table 5**). Most clusters, including *OFP2*, *OFP4*, and *OFP17*, displayed unimodal *Ka/Ks* distributions with values less than 1, consistent with purifying selection and high sequence stability. All intra-accession homologous gene pairs of OFP across the 17 accessions exhibited *Ka/Ks* < 1 (**Figure 4B**), consistent with purifying selection at the sequence level. Collectively, purifying selection has played a predominant role in OFP evolution, whereas a minority of members with elevated pairwise dN/dS ratios may reflect accession-specific adaptive differentiation.

**Figure 4:**
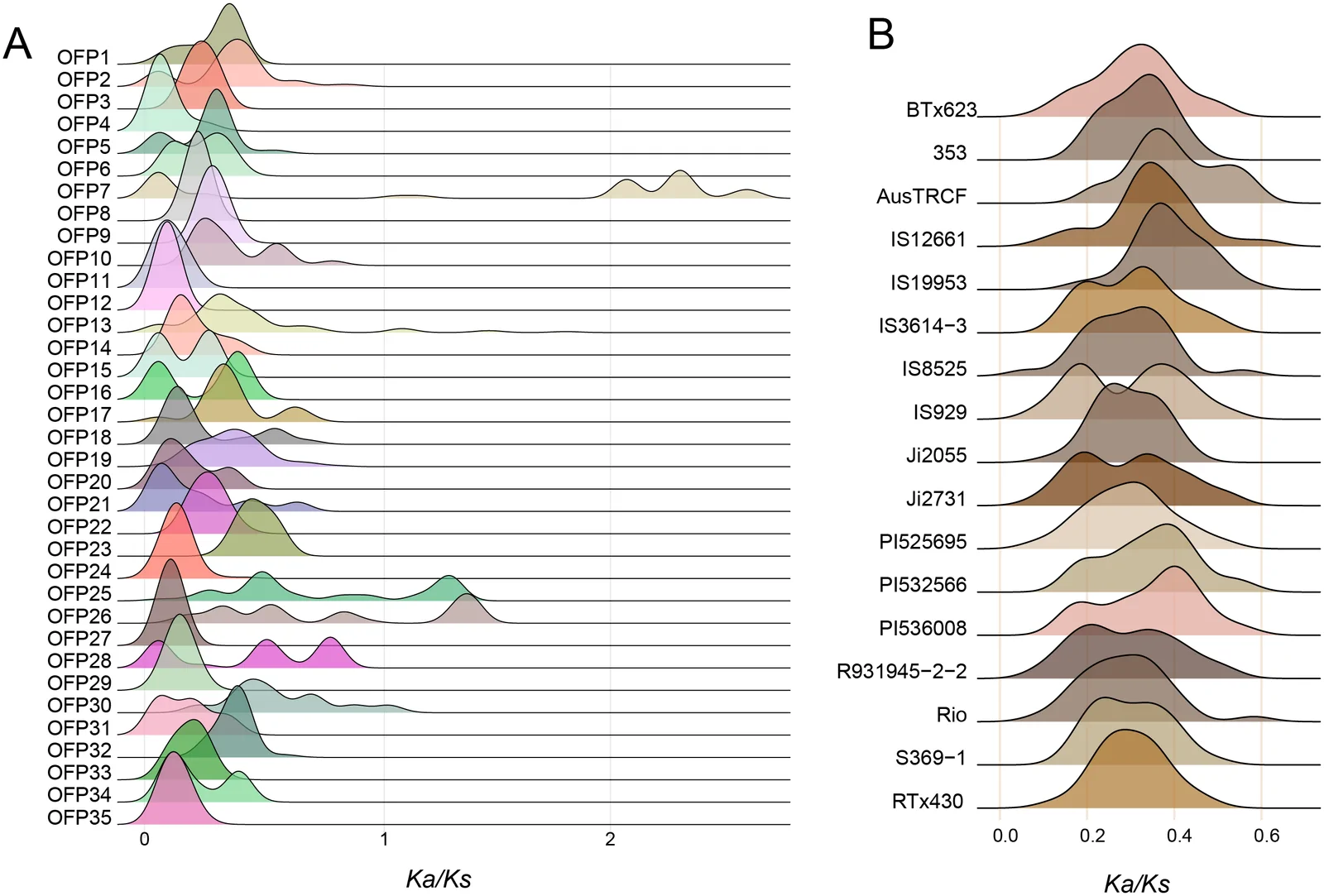
Distribution of Ka/Ks values for OFP genes across 17 sorghum accessions. **(A)** Ka/Ks values of paralogous SbOFP gene pairs (segmental/tandem duplicates) across the 17 sorghum accessions, grouped by orthologous family. **(B)** Ka/Ks values of collinear SbOFP gene pairs identified by intra-accession collinearity (synteny) analysis within individual accession genomes.

### 3.4 Cis-acting element analysis of *SbOFP* promoters

Cis-acting element prediction was performed for the promoter regions of 498 *SbOFP* genes identified from 17 sorghum genomes. In total, 21,046 cis-elements were detected and classified into phytohormone-responsive elements, stress-responsive elements, and growth/development-related regulatory elements (**Figure 5**). Circadian rhythm-related elements were the most abundant, with 4,578 occurrences (21.8%), followed by MeJA-responsive elements (4,460, 21.2%), suggesting that *SbOFP* genes are involved in circadian rhythm and jasmonate signaling regulation. Promoters of *SbOFP* genes also contained elements associated with meristem expression, seed specificity, and endosperm-related expression, implying potential roles in grain development.

**Figure 5:**
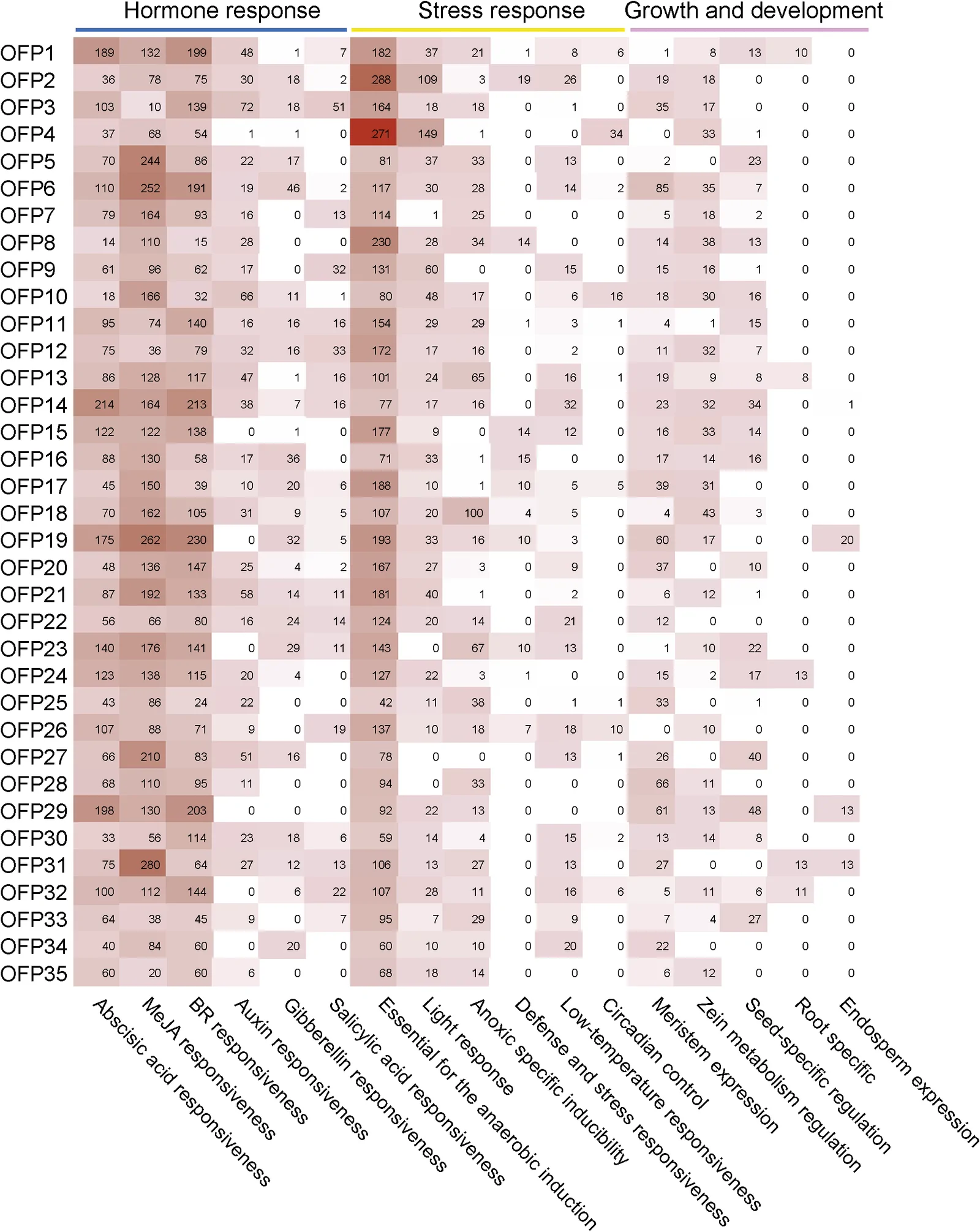
Distribution of cis-acting elements in the promoter regions of sorghum OFP genes. The 2-kb upstream sequences of all 498 SbOFP genes from the 17 accessions were analyzed. Element counts were aggregated across all promoters of each orthologous family.

Element counts were calculated as the total across all promoters of each orthologous family and therefore represent family-level motif abundance. Sorghum OFP family member promoters contained multiple predicted hormone-responsive motifs, including those responsive to abscisic acid (ABA), brassinosteroids (BR), auxin (AUX), gibberellin (GA), and salicylic acid (SA), suggesting coordinated regulation of *SbOFP* expression by multiple hormonal pathways (**Figure 5**). ABA-responsive elements were widely distributed across the family. Promoters of *OFP14* contained the highest number of ABA-related elements (214) and also harbored 164 BR-responsive and 213 MeJA-responsive elements, representing the highest overall abundance of these motifs among all family members. *OFP2* showed the greatest total number of BR-responsive elements (288), followed by *OFP31* (280) and OFP5 (252). *SbOFP* genes exhibited features consistent with multi-hormone responsiveness. Among these, *OFP6* was simultaneously enriched for BR-responsive (191), MeJA-responsive (252), and ABA-responsive (110) elements, displaying high promoter densities of hormone-responsive motifs. In contrast, other members showed hormone-specific preferences, as exemplified by *OFP5* and *OFP29*, with promoters containing markedly higher abundances of specific hormone-related elements than most other genes. Additionally, BR-responsive G-box motifs in *OFP3* (139) outnumbered its GA-responsive elements (72).

Light-responsive elements were broadly detected across the family. *OFP8* contained the largest number of light-responsive elements (230). Elements associated with anaerobic induction, low-temperature response, and defense-specific induction were also identified with gene-specific distribution patterns. The *OFP4* promoter was highly enriched for anaerobic induction elements (271), while *OFP18* contained abundant defense-specific inducible elements (100), suggesting potential involvement of *SbOFP* genes in both abiotic and biotic stress responses. The distribution of growth- and development-related elements further reflected the functional features of *SbOFP* genes. Meristem expression elements were enriched in *OFP5*, ranging from 5 to 7 elements per gene with an average of 6.13. In addition, *SbOFP* members contained seed-specific and endosperm-related expression elements, supporting their role in grain development (**Figure 5**).

### 3.5 Expression analysis of *OFP* genes to exogenous hormone treatment

To elucidate the hormone-responsive characteristics of *SbOFP* genes, RT-qPCR was performed to analyze expression changes following foliar application of BR, MeJA, ABA, and IAA. The promoter regions of *SbOFP* genes in the BTx623 reference genome contained varying numbers of hormone-responsive elements (**Figure 6A**), and the hormone treatments were performed on BTx623. OFP6 harbored the highest number of BR-responsive elements (16), followed by *OFP14* (12). *OFP33* contained 22 MeJA-responsive elements, the highest abundance among all family members, while *OFP2* contained 8 MeJA-responsive elements. *OFP13* possessed the greatest number of ABA-responsive elements (16), and *OFP4* and *OFP10* had the highest counts of auxin-responsive elements (9 and 6, respectively).

**Figure 6:**
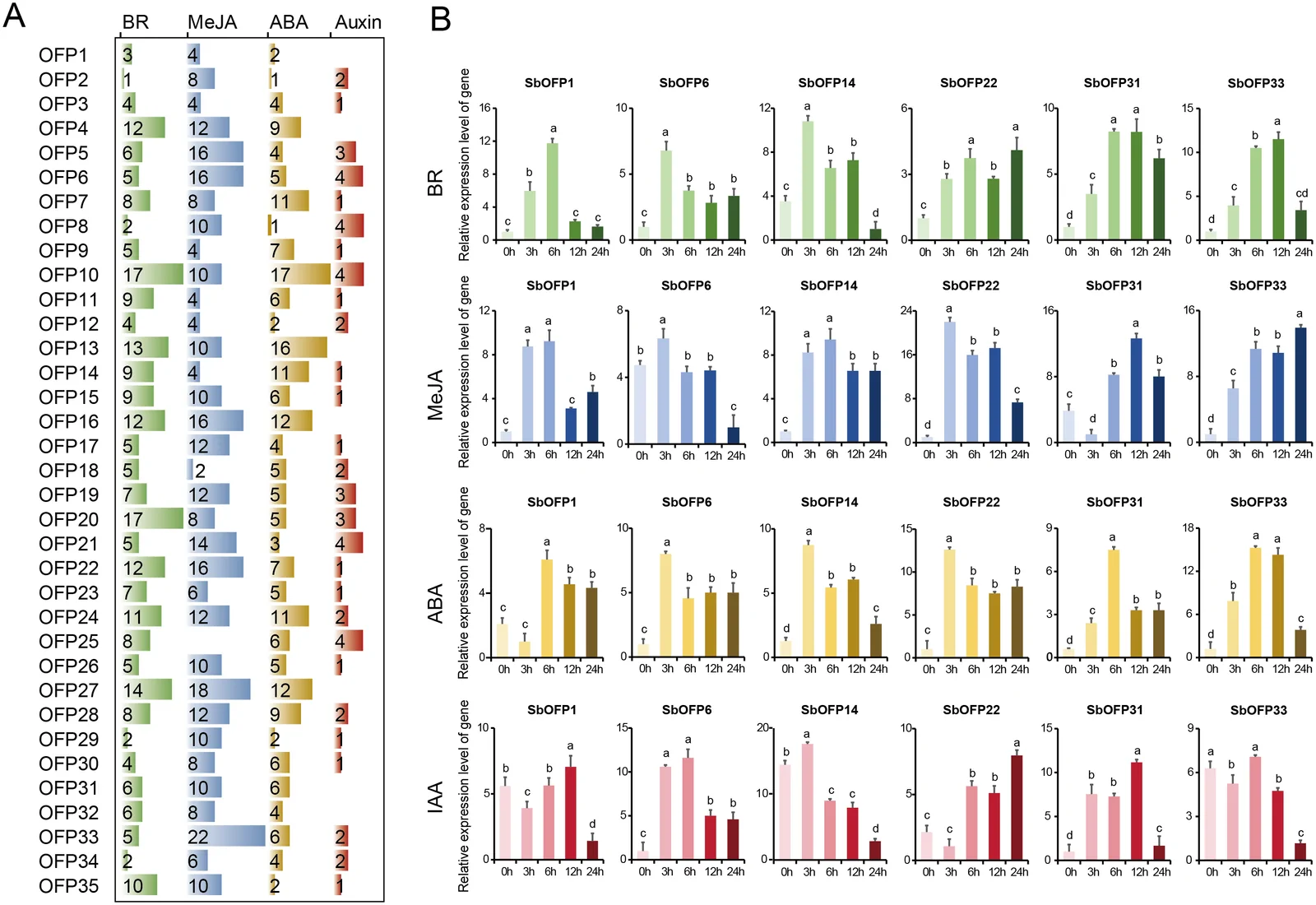
Hormone-responsive expression patterns of SbOFP genes. **(A)** Number of hormone-responsive cis-acting elements in the promoter regions of 35 SbOFP genes. BR, brassinosteroid, MeJA, methyl jasmonate, ABA, abscisic acid, IAA, indole-3-acetic acid. **(B)** Relative expression levels of SbOFP genes at 0, 3, 6, 12, and 24 h following treatment. Data are means ± SD (n = 3 biological replicates). Different letters indicate significant differences (two-way ANOVA followed by Tukey’s HSD test, *p* < 0.05).

Six representative genes were selected for expression analysis based on their distinct element compositions. Under BR treatment, *SbOFP1* and *SbOFP6* peaked at 3 h, while *SbOFP14*, *SbOFP22*, and *SbOFP31* peaked at 6 h; *SbOFP33* peaked at 12 h. Under MeJA treatment, *SbOFP1*, *SbOFP6*, *SbOFP14*, and *SbOFP22* all peaked at 3 h, whereas *SbOFP31* and *SbOFP33* peaked at 6 h and 12 h, respectively. For ABA treatment, *SbOFP1*, *SbOFP6*, and *SbOFP14* peaked at 3 h; *SbOFP22* at 6 h; and *SbOFP31* and *SbOFP33* at 12 h. Under IAA treatment, *SbOFP1* and *SbOFP6* showed continuous increases and peaked at 12 h, while *SbOFP14*, *SbOFP22*, and *SbOFP31* peaked at 6 h. Notably, *SbOFP33* exhibited a rapid response to IAA, reaching its maximum at 3 h (**Figure 6B**).

These differential hormone response patterns were consistent with the distribution of cis-acting elements. *SbOFP6*, which contained abundant BR-responsive elements (16), showed strong early induction by BR. *SbOFP33*, which harbored the highest number of MeJA-responsive elements (22), exhibited a marked response to MeJA despite its delayed peak. *SbOFP14*, enriched for both BR- (12) and ABA-responsive (4) elements, displayed rapid responses to these hormones. *SbOFP22*, with substantial BR- (7), MeJA- (16), and ABA-responsive (7) elements, peaked early under BR and MeJA. *SbOFP31*, containing considerable BR- (10) and IAA-responsive (2) elements, exhibited sustained expression under both treatments. *SbOFP* gene expression showed significant changes following BR, MeJA, ABA, and IAA treatments. These differential responses were associated with promoter element composition, suggesting the functional relevance of these motifs in hormone-responsive transcriptional regulation. We note that the seedling-leaf responses observed here cannot be directly extrapolated to grain development.

### 3.6 Expression characteristics and interaction networks of OFP genes during sorghum grain development

To clarify the expression characteristics of *SbOFP* genes during grain development, expression levels were analyzed in two cultivated sorghum varieties, HN16 (small-grain) and QS (large-grain), at 8, 14, and 28 days after anthesis (DAP). The OFP gene family exhibited significant stage specificity and varietal divergence. *SbOFP14* and *SbOFP15* maintained relatively high expression levels throughout all stages, and were identified as dominantly expressed members during grain development. *SbOFP21* and *SbOFP23* showed elevated expression at 14 DAP that subsequently declined, whereas *SbOFP29* showed elevated expression at 14 DAP, followed by a decline at 28 DAP (**Figure 7A**). Collectively, the majority of OFP members showed high expression at the middle stage of grain development (14 DAP).

**Figure 7:**
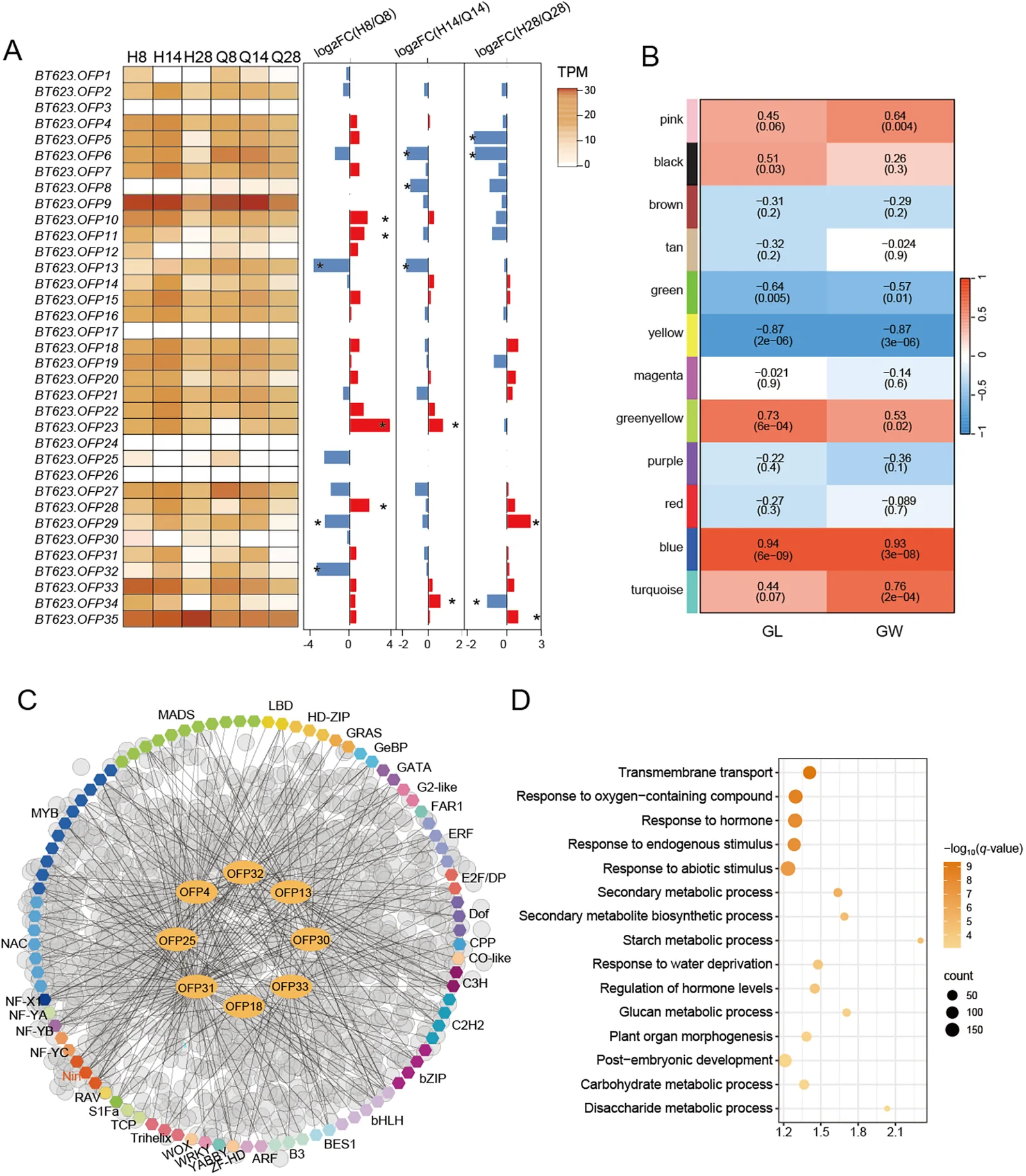
Expression characteristics and co-expression network analysis of SbOFP genes during grain development. **(A)** Differential expression of SbOFP genes between varieties. Left panel: TPM expression levels of SbOFP genes in HN16 and QS at 8, 14, and 28 DAP. Right panels: log2 fold change of gene expression between HN16 and QS at corresponding stages. Blue bars indicate higher expression in QS, red bars indicate higher expression in HN16. *adjusted *p* < 0.05. **(B)** Module-trait correlation heatmap showing correlations between WGCNA modules and grain length (GL) and grain width (GW). **(C)** Co-expression network of SbOFP hub genes and transcription factors. **(D)** GO enrichment analysis of blue module genes.

Genetic background significantly influences *OFP* gene expression. Across the three periods, the HN16 variety demonstrated higher *OFP* expression levels, with *SbOFP3*, *SbOFP14*, and *SbOFP15* showing significantly elevated expression in HN16 compared to QS. RT-qPCR validation was performed on 6 *SbOFP* genes with high expression in grains, and the results confirmed that the expression trends of these genes were consistent with transcriptomic data (**Supplementary Figure 3**).

Based on grain transcriptome data, 20,120 filtered genes were used for WGCNA, identifying 12 co-expression modules. The blue module, which comprised 4,200 genes, showed a significant positive correlation with grain length and width (*r* > 0.8, *p* < 0.01) (**Figure 7B**; **Supplementary Figures 4, S5**). As grain length and width increase with developmental progression, this module–trait correlation may partly reflect developmental stage; the blue module is interpreted as grain-development-associated. Within this module, 436 genes exhibited high intramodular connectivity (degree > 30) and were defined as hub genes, among which 8 were SbOFP genes. These SbOFPs were used as target genes for subsequent regulatory network. Given that OFP proteins lack typical DNA-binding domains and indirectly exert transcriptional repressor activity through interactions with DNA-binding transcription factors, we employed the bottom-up GGM algorithm to construct an ML-hGRN with *SbOFP* hub genes as the basal-layer target genes and candidate transcription factors (TFs) as the upper regulatory layer. This ML-hGRN comprised 34 transcription factor families in the second layer, including NAC, MYB, MADS, LBD, HD-ZIP, and bHLH, which showed significant interactions with target *SbOFP* genes. These TFs represent candidate regulators of *SbOFP* genes (**Figure 7C**). Among the *SbOFP* hub genes, *SbOFP13* and *SbOFP32* showed the highest connectivity with TFs in the network.

GO enrichment analysis revealed that genes in the blue module, including *SbOFPs* and their TF partners, may regulate grain size by coordinating hormone signaling pathways, stress response, and carbohydrate metabolism during grain filling, representing key candidate genes for grain development (**Figure 7D**; **Supplementary Table 8**). These findings suggest that OFP proteins may function as transcriptional co-regulators through association with these TFs (**Figure 7D**).

## 4 Discussion

### 4.1 Genomic evolution of the sorghum OFP family reflects conserved core functions and adaptive diversification

Pan-genome analysis provides a comprehensive strategy for capturing gene family variation that cannot be resolved using a single reference genome (Tao et al., 2021). In the present study, a total of 498 *OFP* genes were identified across 17 sorghum accessions, with each genome containing 19-36 members (mean 29). This relatively narrow range suggests that the OFP family maintains a stable genomic architecture across diverse sorghum germplasm, consistent with the pattern of conserved core genes observed in pan-genome analyses of other crops (Mace et al., 2013). Five Core families were retained across all accessions, with OFP4, OFP5, and OFP6 maintained as strict single-copy genes, and with average amino acid identity of 96.5% and *Ka/Ks* values consistently below 1, indicating strong purifying selection. This pattern was similar to the evolutionary features of near-core genes in other species. In tea plants, near-core YABBY genes similarly exhibit purifying selection and structural stability (Wen et al., 2026). Notably, the consistent retention of these core OFP genes across all accessions implies that they may be indispensable for normal development. The absence of Private OFP families likely reflects the ancient origin and strong functional constraint of this family, as the same clustering pipeline readily detected Softcore and Shell families and copy-number variation.

In contrast, Shell genes accounted for over half of total OFP genes (54.0%) and showed greater diversity. OFP1, OFP9, and OFP31 exhibited copy number variation with 2–3 copies in specific accessions, reflecting small-scale duplication events during sorghum diversification. Shell OFPs generally possessed more diverse protein sizes, molecular weights, and distinct physicochemical properties compared to core members, suggesting they may have acquired new functional roles during evolution (Han et al., 2022). The high proportion of Shell genes and their association with specific accessions imply that these variable members may contribute to adaptive differentiation among sorghum ecotypes, potentially responding to diverse environmental conditions encountered during the spread of cultivated sorghum (Tao et al., 2021).

Collinearity analysis revealed that syntenic relationships of OFP genes were highly conserved within *S. bicolor* ssp. *bicolor* accessions, whereas fewer syntenic connections were detected between subspecies. This gradient reflects the phylogenetic relationships among the 17 accessions, where wild “*S. propinquum*” and “*S. bicolor* ssp. *verticilliflorum*” represent early-diverged lineages, while cultivated “*S. bicolor* ssp. *bicolor*” forms a tight cluster (Tao et al., 2021; An et al., 2024). The evolutionary dynamics of the OFP family are thus closely associated with the genetic relatedness of sorghum germplasm. We speculate that the stable retention of Core OFPs across all accessions reflects their indispensable roles in fundamental developmental processes, while the expansion and diversification of Shell genes may represent functional diversification of OFP to adapt to diverse germplasm characteristics.

### 4.2 Hormone-responsive expression patterns of *SbOFP* genes

Cis-acting element analysis of 498 *SbOFP* promoters identified abundant regulatory elements, with hormone-responsive motifs being particularly prevalent. The promoters contained elements responsive to ABA, BR, auxin, GA, MeJA, and SA, indicating that *SbOFP* expression is coordinately regulated by diverse hormonal pathways. This pattern was consistent with findings in cucumber, where a series of cis-elements related to hormone responsiveness were identified in the promoter regions of *CsOFP* genes (Han et al., 2022). In poplar, ABRE, TGA-element, and TGACG-motif were distributed in *PtOFP* promoter regions, indicating participation in IAA, GA, ABA, SA, and MeJA-related processes (Wang et al., 2021). Similarly, hormone-responsive cis-elements were abundantly identified in *Brassica napus OFP* promoters, including motifs for ABA, GA, and auxin signaling (Liu et al., 2022), indicating hormonal regulation of OFP genes as a conserved feature across diverse plant species. These findings suggest that OFPs may differentially regulate plant architecture and grain development through distinct hormonal pathways.

The functional relevance of these cis-elements was supported by exogenous hormone treatments. *SbOFP1*, containing BR (3), MeJA (4), and ABA (2) responsive elements, showed rapid induction by these three hormones and peaked at 3 h. *SbOFP6* contained the most BR-responsive elements (16) and showed strong early induction by BR. These distinct response patterns were consistent with observations in rice, where *OsOFP2* contains cis-acting elements associated with ABA and GA synthesis and interacts with KNOX and BELL proteins to regulate vascular bundle development (Schmitz et al., 2015). Similarly, *OsOFP1* positively regulates BR signaling by interacting with core BR components DLT, OsBZR1, and GSK2 kinase, and its overexpression produced plants with reduced plant height, enlarged leaf angles, and altered grain shape (Xiao et al., 2017). Distinct rice OFP members play opposing roles in BR signaling, as OsOFP8 positively mediates BR signaling through interaction with the GSK3-like kinase (Yang et al., 2016). OsOFP3 acts as a suppressor of BR responses and is stabilized by GSK2-mediated phosphorylation (Xiao et al., 2020). In sorghum, the differential hormone response patterns of *SbOFP* genes suggest that individual family members may serve as regulators of hormone signaling during sorghum development.

Tissue-specific expression profiling revealed that most *SbOFP* genes were preferentially expressed in reproductive tissues, particularly during seed development. *SbOFP14* and *SbOFP15* maintained high expression throughout all grain developmental stages. This pattern was consistent with banana *MaOFP1*, which is predominantly expressed in flowers and fruits and interacts with MaMADS36 and hormone response proteins to regulate fruit development and ripening (Zhang et al., 2020). In banana, *MaMADS1* and *MaOFP1* are antagonistically regulated by ethylene, and their physical interaction through the K-domain and OVATE-domain suggests that MADS-OFP complexes represent a conserved regulatory module across species (Liu et al., 2015).

Varietal comparison between large-grain and small-grain sorghum revealed significant differential expression for *SbOFP* genes. *SbOFP3*, *SbOFP14*, and *SbOFP15* were significantly upregulated in HN16, with *SbOFP3* showing the most pronounced divergence during early grain development. This genotype-dependent pattern suggests that OFP genes contribute to grain size variation in a genetic background-specific manner, consistent with rice where *OsOFP9* directly interacts with BR regulators GS9 and DLT to coordinately regulate grain size by opposing their transcriptional activities (Lu et al., 2025). We speculate that differential expression of sorghum OFP members among varieties may reflect allelic variation in promoter cis-elements that modulates hormone responsiveness, thereby influencing grain filling rate and final grain size through altered cell proliferation or expansion.

### 4.3 Co-expression networks and functional mechanisms

A distinctive feature of the OFP family is the absence of typical DNA-binding domains (Snouffer et al., 2020). Transcriptional regulatory activity through protein-protein interactions has been widely demonstrated in multiple studies. In *Arabidopsis*, AtOFP1 interacts with ATH1 to regulate the transition to flowering and modulates gibberellin homeostasis through transcriptional repression of AtGA20ox1 (Wang et al., 2007; Zhang et al., 2018). AtOFP4 participates in regulating secondary cell wall formation through interaction with the BLH6-KNAT7 complex (Liu and Douglas, 2015). In rice, OsOFP1 interacts with DLT and GSK2 to modulate BR signaling and grain morphology (Xiao et al., 2017). OsOFP6 binds to class I KNOX protein OSHB15 and upregulates *OSHB15* expression (Sun et al., 2020). In sorghum, WGCNA identified the blue module as significantly associated with grain size traits. This module contained 8 *SbOFP* genes, whose hub members established extensive co-expression relationships with transcription factors from the NAC, MYB, MADS, LBD, HD-ZIP, and bHLH families. The involvement of these TF families is consistent with their established functions in grain development (Long et al., 2024). NAC and MYB transcription factors regulate secondary cell wall biosynthesis and stress responses (Nakano et al., 2015; Yogendra et al., 2017), while MADS-box genes control floral organ identity and seed development (Gramzow and Theissen, 2010; Zhang et al., 2024).

Given that OFP proteins lack DNA-binding domains, we employed the bottom-up GGM algorithm to construct an ML-hGRN with *SbOFP* hub genes as target genes and candidate TFs constituting the regulatory layer (Kumari et al., 2016). The ML-hGRN approach offers distinct advantages for identifying transcriptional regulators of non-DNA-binding proteins such as OFPs. This method specifically identifies regulatory genes that significantly interfere with the target genes, thereby distinguishing direct regulatory connections from indirect associations. For OFP proteins, which function as transcriptional co-repressors through protein-protein interactions rather than direct DNA binding, the ML-hGRN framework provides a biologically meaningful strategy to pinpoint candidate DNA-binding TFs that may act upstream of SbOFP genes.

The TFs identified through ML-hGRN analysis in this study share notable commonalities with those reported in previous OFP functional studies across species (Zhan et al., 2025). AtOFP1 interacts with BLH/ATH1 TALE homeodomain proteins, while AtOFP4 forms complexes with KNAT7 and BLH6 (Wang et al., 2007; Liu and Douglas, 2015). Similarly, rice OsOFP1 physically associates with the BR signaling components DLT and OsBZR1, and OsOFP6 binds to OSHB15, a class I KNOX protein (Xiao et al., 2017; Sun et al., 2020). The NAC, MYB, MADS, LBD, HD-ZIP, and bHLH families identified in our sorghum ML-hGRN overlap substantially with these functionally validated interaction partners. Notably, the TALE homeodomain and MADS-box families appear recurrently across *Arabidopsis*, rice, and sorghum studies (Tan et al., 2021), suggesting that these TF families are candidate interaction partners of OFP proteins.

These findings support the reliability of the ML-hGRN predictions (Kumari et al., 2016), and underscore a conserved functional paradigm: OFP proteins serve as modular transcriptional repressors that are recruited by diverse DNA-binding TFs to specific genomic contexts, thereby regulating developmental and metabolic processes. Co-expression of *SbOFP* hub genes with these families, together with GO enrichment analysis, suggests that OFPs may regulate grain size by influencing cell wall properties, hormone signaling, and coordinating carbohydrate metabolism during grain filling. The stable conservation of OFP genes across diverse germplasm, together with the discovery of specific expression patterns associated with grain development (Xiao et al., 2020; Lu et al., 2025), provides candidate genes for future functional validation and sorghum breeding. The WGCNA and ML-hGRN analyses were based on 18 samples; the resulting networks should therefore be regarded as hypothesis-generating and require experimental validation.

## 5 Conclusions

A pan-genome analysis of 17 sorghum accessions identified 498 SbOFP genes classified into 35 orthologous gene families, comprising 5 Core, 9 Softcore, and 21 Shell families. Core OFP genes were consistently retained across all accessions (at least one copy per genome) and exhibited significantly higher sequence stability under purifying selection compared to Shell members. Two Softcore (OFP7, OFP13) and three Shell (OFP25, OFP26, OFP30) members exhibited elevated pairwise Ka/Ks (dN/dS) ratios in subsets of inter-accession comparisons. Expression profiling revealed predominant SbOFP expression in seed and inflorescence tissues, with *SbOFP14* and *SbOFP15* identified as dominantly expressed members throughout grain development. Promoter analysis detected abundant hormone-responsive and grain development-related cis-acting elements, and exogenous hormone treatments showed that SbOFP genes exhibited expression changes following ABA, BR, MeJA, and IAA treatments, with response patterns broadly consistent with the presence of corresponding responsive elements in their promoters. Weighted gene co-expression network analysis further revealed that hub SbOFP genes, particularly SbOFP32 and SbOFP13, are co-expressed with NAC, MYB, MADS, and bHLH transcription factors, which represent candidate partners of SbOFP genes during grain development. These findings provide candidate genes and testable hypotheses for future studies on SbOFP functions in grain development.

## Data Availability

The transcriptome sequencing data generated in this study have been deposited in the China National GeneBank DataBase (CNGB) under the BioProject accession number PRJCA066477.
