# Supplementary material for: Pangenomic characterization and grain-development expression analysis of the OVATE gene family in sorghum
Source: Front Plant Sci. 2026 Sep 17;17:1926574. doi: 10.3389/fpls.2026.1926574 (PMC13627345; doi:10.3389/fpls.2026.1926574)
Supplement: Supplementary file 1 [file DataSheet1.docx]

**Pangenomic characterization and grain-development expression analysis of the OVATE gene family in sorghum**

**Yukun Gao^1^, Puyuan Yang^1^, Yongliang Han^2^, Weiping Yang^1^, Xiaodong Xiang^3*^, and** **Jinhua Chang^1*^**

^1^Key Laboratory of Crop Germplasm Resources in North China, Ministry of Education, College of Agronomy, Hebei Agricultural University, Baoding, China,

^2^Handan Academy of Agricultural Sciences, Handan, China,

^3^Key Laboratory of Biodiversity Conservation and Sustainable Utilization in Mongolian Plateau for College and University of Inner Mongolia Autonomous Region, College of Life Science and Technology, Inner Mongolia Normal University, Hohhot, China

*** Correspondence:**

Jinhua Chang
[jhchang2006@126.com](mailto:jhchang2006@126.com)

Xiaodong Xiang

[xiangxd@imnu.edu.cn](mailto:xiangxiaodong@imnu.edu.cn)

**FIGURE S1** Phylogenetic Analysis of the Sorghum Pangenomic OFP Family and *Arabidopsis* OFP Genes

**FIGURE S2** Chromosomal Distribution of the OFP Gene Family in *Sorghum bicolor* cv. BTx623

**FIGURE S3** RT-qPCR validation of RNA-seq expression profiles for six *SbOFP* genes.

**FIGURE S4** Sample clustering and weighted gene co-expression network analysis (WGCNA) of sorghum grain transcriptome data. (**A**) Hierarchical clustering dendrogram of all RNA-seq samples (B) Cluster dendrogram of genes and corresponding module assignment.

**FIGURE S5** Expression dynamics of the blue module during grain development.
